# Fourier Analysis of Conservation Patterns in Protein Secondary Structure

**DOI:** 10.1016/j.csbj.2017.02.002

**Published:** 2017-02-22

**Authors:** Ashok Palaniappan, Eric Jakobsson

**Affiliations:** a Dept of Biotechnology, Sri Venkateswara College of Engineering, Post Bag No. 3, Pennalur, Sriperumbudur 602117, India; b University of Illinois at Urbana–Champaign, IL 61820, USA

**Keywords:** Periodicity, Secondary structure, Evolution, Moment of conservation, Fourier transform, Potassium channel

## Abstract

Residue conservation is a common observation in alignments of protein families, underscoring positions important in protein structure and function. Though many methods measure the level of conservation of particular residue positions, currently we do not have a way to study spatial oscillations occurring in protein conservation patterns. It is known that hydrophobicity shows spatial oscillations in proteins, which is characterized by computing the hydrophobic moment of the protein domains. Here, we advance the study of moments of conservation of protein families to know whether there might exist spatial asymmetry in the conservation patterns of regular secondary structures. Analogous to the hydrophobic moment, the conservation moment is defined as the modulus of the Fourier transform of the conservation function of an alignment of related protein, where the conservation function is the vector of conservation values at each column of the alignment. The profile of the conservation moment is useful in ascertaining any periodicity of conservation, which might correlate with the period of the secondary structure. To demonstrate the concept, conservation in the family of potassium ion channel proteins was analyzed using moments. It was shown that the pore helix of the potassium channel showed oscillations in the moment of conservation matching the period of the *α*-helix. This implied that one side of the pore helix was evolutionarily conserved in contrast to its opposite side. In addition, the method of conservation moments correctly identified the disposition of the voltage sensor of voltage-gated potassium channels to form a 3_10_ helix in the membrane.

## 1 Introduction

Amino-acid conservation is an evolutionary property. Physical properties of amino acid side-chains exhibit a higher-order moment (also known as periodicity) in the context of repetitive secondary structures, such as the *α*-helix and *β*-sheet. A notable physical property whose moments turned out to be significant is the hydrophobicity [1]. The disposition of structured domains in the protein is strongly correlated with the overall hydrophobicity and the amphiphilicity of the domains. These properties stabilize the structure of the protein and, for membrane proteins, the protein's association with the membrane. For *α*-helical membrane proteins, the strength of the hydrophobic moment is maximal at the period of the helix (i.e, 100°). Similarly, for beta-barrel membrane proteins, the hydrophobic moment is maximal at the period of the beta sheet (i.e, 160°–180°). In both cases, the surface of the secondary structure element which is in contact with lipid exhibits a strong hydrophobicity to allow for partitioning into the membrane. Domains exposed to the electrolyte on either side of the membrane exhibit strong amphiphilicity, or a high hydrophobic moment. The periodicity of residue properties of *α*-helical proteins can be visualized using the helical wheel representation. Spatial asymmetry in the distribution of hydrophobicity, say, on the helical wheel would imply the fine-tuning of protein function via the achievement of amphiphilicity. Amphiphilicity has turned out to be key to the activity of antimicrobial peptides. Most native and engineered antimicrobial peptides face the amphiphilicity requirement to successfully insert into and permeabilize the bacterial membrane [2].

The use of sequence profiles improved the ability of hydrophobicity to predict the formation of *α*-helices [3]. Analogous to hydrophobicity, we consider that the residue conservation in a protein alignment displays a first-order moment. Residue conservation is directly correlated with general functional importance. The moment of residue conservation would likely contain information not captured by a linear residue-by-residue conservation. The evolutionary basis of the moment of conservation is as follows: one face of an *α*-helix involved in critical interatomic interactions must be conserved, while the diametric face might not be equally constrained and evolve with neutral drift. In order to detect and quantify this spatially oscillatory constraint in the protein secondary structure, we introduce a measure called ‘conservation moment’ and illustrate its applications.

## 2 Material and Methods

### 2.1 Calculation of the Zeroth Moment of Conservation

The zeroth moment of conservation is the sum of the conservation values of the residues based on a profile of homologous sequences. The profile is built using homology detection methods and multiple sequence alignment. The conservation *c*_*n*_ of each column *n* of the alignment could then be computed using, for e.g., Shannon entropy:

(1) $$c_{n}=-\underset{i}{\sum }p_{i}\ln p_{i}$$

where the *p*_*i*_’s are the probabilities of finding residue *i* in column *n* and the summation is over all the 20 amino acids. The *c*_*n*_’s are scaled from 0 to 1, 0 denoting a column of all different residues and 1 denoting a column of all identical residues. The resulting one-dimensional function of conservation values over the length of the alignment is called the conservation vector. The zeroth conservation moment *C*_0_ of an alignment segment of length *N* is equal to the sum of the *c*_*n*_’s of the columns of the alignment segment.

(2) $$C_{0}=\overset{N}{\underset{n=1}{\sum }}c_{n}$$

*C*_0_ is a measure of the net conservation of an alignment segment. A contiguous sequence of conserved residues in a protein family would give rise to a high *C*_0_.

### 2.2 Calculation of First-order Conservation Moment

To detect an asymmetry in the conservation pattern of an alignment segment, we search for periodicities in the corresponding conservation vector. The moment of the conservation vector at a given periodicity is a measure of the signal strength at that periodicity, and is known as the first-order conservation moment, *C*_1_(*θ*). For a given period *θ*,

(3) $$C_{1}(\theta )={\left\{{\left[\overset{N}{\underset{n=1}{\sum }}C_{n}\sin\;(\theta n)\right]}^{2}+{\left[\overset{N}{\underset{n=1}{\sum }}C_{n}\cos\;(\theta n)\right]}^{2}\right\}}^{\frac{1}{2}}$$

where *N* is the length of the alignment segment, and the period *θ* is measured in radian. An evolutionary asymmetry in the *α*-helix structure would be manifested as a strong conservation moment at the period of the *α*-helix. This corresponds to *θ* = 2*π*/100° = 3.6 rad. Similarly, an evolutionary moment in the *β*-sheet structure would give rise to a maximal signal at the period of the *β*-sheet (=160°–180°). Eq. (3) could be rewritten as the modulus of the fourier transform of the conservation vector.

(4) $$C_{1}(\theta )=\left|\overset{N}{\underset{n=1}{\sum }}C_{n}e^{i\theta n}\right|$$

## 3 Results and Discussion

When the protein secondary structure is known, from a crystal structure or otherwise, *C*_1_(*θ*) could be calculated for each secondary structure element at its respective period to detect any spatial asymmetry in evolutionary pressure. Periodicity in evolutionary pressure is valuable for transmembrane structures which accommodate hydrophobic constraints to be stable in the lipid bilayer. This might enable the transmembrane structure to achieve a higher-order functional specificity. An illustrative secondary structure element is the pore helix of the potassium ion (*K*^ +^) channel.

Potassium channels are tetrameric transmembrane (TM) structures with two TM helices per subunit [6]. In addition, each subunit has a pore helix that spans half the membrane before looping back. These pore-helices are under an interesting evolutionary constraint. By virtue of scaffolding the ‘selectivity filter’ of potassium channels, their packing interfaces are evolutionarily constrained. This sidedness of conservation could be detected using the first-order conservation moment. A profile of all the human potassium channel sequences was constructed in the following manner. A representative sequence of each potassium channel subfamily was chosen, and used as a query in PSI-BLAST with an E-value of 0.001 until convergence [7]. After eliminating duplicates, each hit was screened for the presence of selectivity filter characteristic of a potassium-selective channel, to obtain 123 channels (available as a supporting information).

Since potassium channels are highly heterogeneous in their domain composition, the permeation pathways of the channels were extracted for further analysis by pivoting about the selectivity filter. It must be noted that the two-pore channels contain two distinct permeation pathways to form a ‘tetramer’ via a hetero-dimer of homo-dimers. After sorting by sequence lengths, multiple rounds of profile–profile alignments were needed to gradually build the global alignment of all human potassium channels [8]. Owing to the variable extracellular turret region, the alignment was manually edited to register the pore-helix and the surrounding TM helices. Finally the KcsA (PDB 1bl8) *K*^ +^-channel sequence was aligned with the rest of the sequences. This alignment was used to calculate the one-dimensional conservation function for each position in the KcsA sequence.

**Fig. 1:**
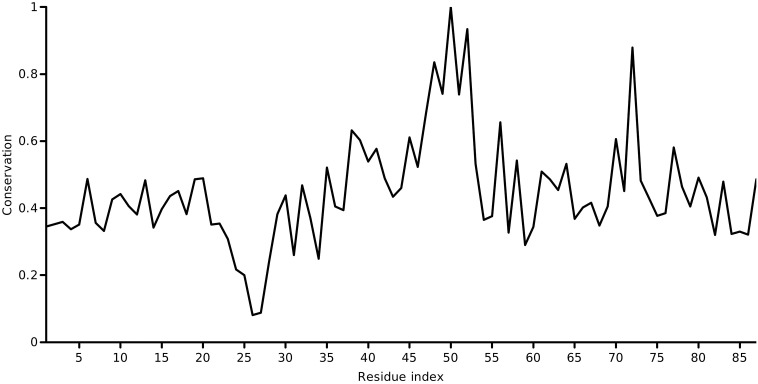
Profile of the conservation of each position in the KcsA potassium channel sequence, as calculated using Scorecons. A peak (conservation = 1.0) corresponding to the selectivity filter of the *K*^ +^-channel could be observed at position 50.

**Fig. 2:**
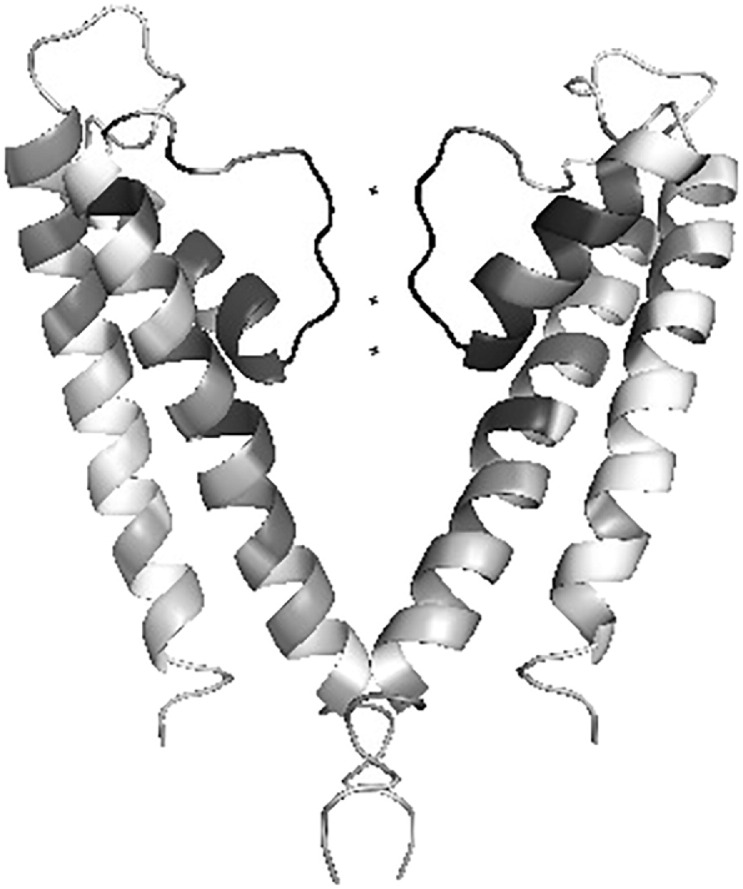
Structural mapping of conservation of human *K*^ +^-channel sequences, using the Consurf algorithm [4]. Imbalance in conservation could be observed. Only two diametric KcsA channel monomers are shown.

Source: Data for this figure obtained from ref. [5].

The conservation of each column of the final alignment was calculated using Scorecons [9], which uses a residue substitution matrix and sequence-weighting to arrive at its final score. These biological refinements to calculating *c*_*n*_ enhance the entropic formulation in Eq. (1). The score for a particular column of the alignment is normalized to the range [0,1] in the order of increasing conservation. Scorecons assessed the informativeness of the alignment and estimated a diversity of 94.6% (higher the diversity, the more informative the alignment). Fig. 1 shows the one-dimensional conservation vector as a function of the residue index of the KcsA channel. The location of the selectivity filter which is essential in establishing the precise selectivity of the channel is evident. A glimpse of the asymmetry in the conservation patterns for the potassium channel could be revealed by mapping the conservation metric of each KcsA residue over its 3D structure. A plot which maps the ‘emphasis' of conservation on the structure is shown in Fig. 2.

**Fig. 3:**
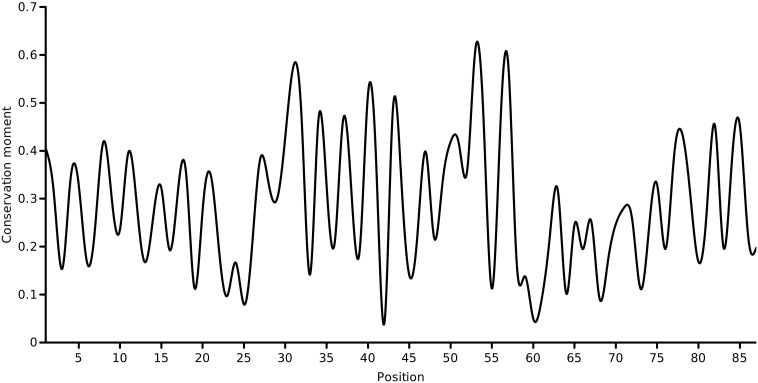
Conservation moment *C*_1_(*θ* = 3.6) of the KcsA potassium channel sequence, computed using Eq. (4) and a sliding window of 11 residues. The pore helix spans positions 35 to 45.

**Fig. 4:**
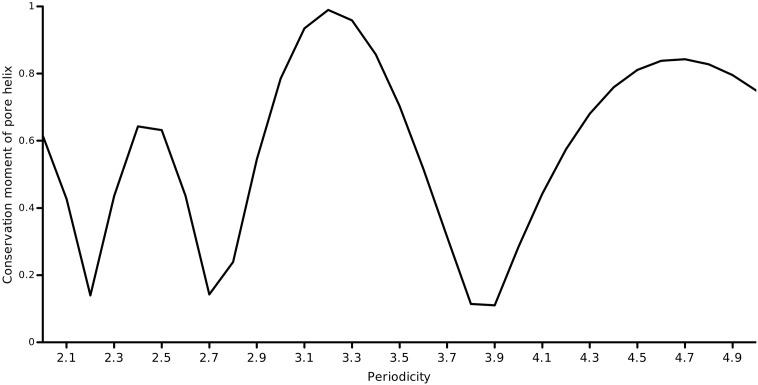
Profile of the conservation moment *C*_1_(*θ*) over the pore helix as a function of periodicity. The conservation moment was estimated about the central residue position of the pore helix using a block size = 11.

**Fig. 5:**
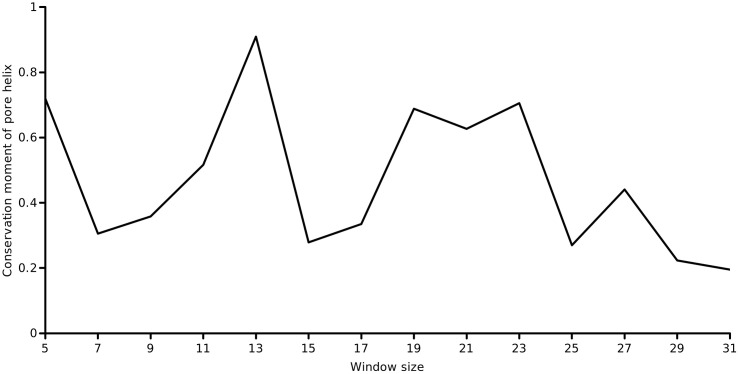
Profile of the conservation moment at *C*_1_(*θ* = 3.6) over the pore helix, as a function of the sliding window size.

The first-order conservation moment, *C*_1_(*θ*), was computed using Eq. 4. The block length was set to the length of the pore helix (=11 residues) and periodicity of interest was the helical periodicity (*θ* = 3.6). Fig. 3 shows the computed conservation moment for a sliding window of 11 residues over the full length of the protein at a periodicity of 100°. This produced a profile of the conservation moment as a function of position (i.e, the center of the sliding window). It was observed that the moment oscillated periodically between low and high values about the pore helix region. These initial observations were in accord with the case for a conservation moment of the pore helix. To investigate whether the period of oscillation coincided with the period of the *α*-helix, we determined the *C*_1_(*θ*) of the pore helix region alone (an ungapped column subset of the alignment of 11 residues) at various periodicities ranging from 2.0 to 5.0 radian in steps of 0.1 radian (shown in Fig. 4). Close to 3.6 radian, it was found that the *C*_1_(*θ*) reached a maximum, validating the helical periodicity of the conservation. To ascertain the variation of *C*_1_(*θ*) with the window size, the window size was varied from 5 to 31 residues around the central residue of the pore helix (default = 11 residues) and the *C*_1_(*θ*)’s at *θ* = 3.6 was computed (shown in Fig. 5). Close to the true length of 11 residues, the maximum moment was observed.

**Fig. 6:**
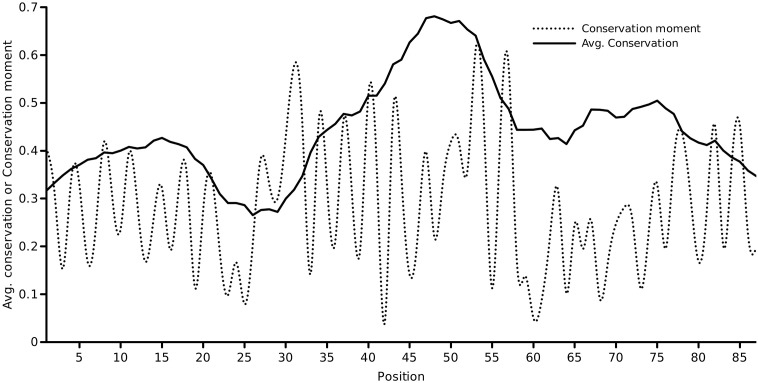
A plot of *C*_1_(*θ* = 3.6) versus the mean conservation (using a window size = 11 residues). In the region from positions 32 to 45, moderate residue conservation is coupled with oscillations in the moment of conservation.

Fig. 6 shows a plot of the conservation moment (*C*_1_(*θ*)) against the normalized net conservation using a sliding window of 11 residues. This is a plot of the first-order moment against the normalized zeroth moment, both defined for a window size = 11. Normalization of the net conservation was achieved by the mean of *C*_0_ for the given length (which is identical to *C*_1_(*θ*) for an infinite period). In the region of the pore helix, a moderate residue conservation coupled with significant oscillations in the moment of conservation could be observed. It appeared that the sidedness property of conservation was more important than the residue conservation *per se*. This would imply that the moment of conservation was more important than the conservation of the identity of the residue.

**Fig. 7:**
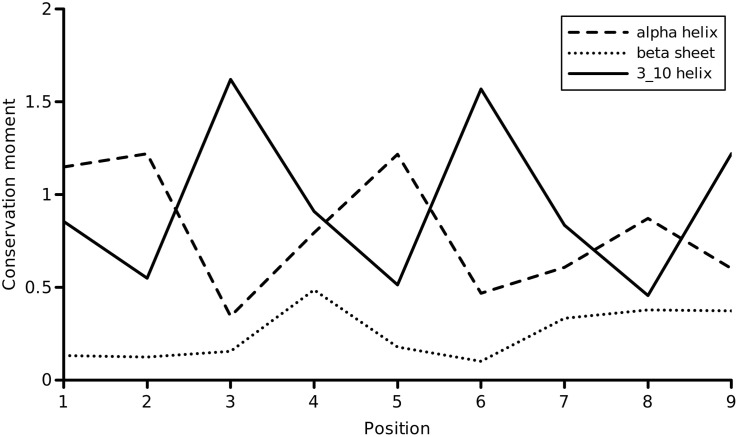
Profile of the conservation moment of the 18-residue voltage sensing module in the S4 transmembrane region of voltage-gated potassium channels, using a window size = 10. Three different secondary structures are probed at their respective periodicities. For the 3_10_ helix, *θ* = 3.0.

To further analyze the utility of the conservation moment, the voltage sensing module of voltage-gated potassium channels was investigated. The voltage sensor is the gating module in ion channels that underlies their steep dependence on membrane voltage for channel opening and closing. The precise mechanism of voltage-gating has remained uncertain [10]. All voltage-gated potassium channels (Kv channels) contain the voltage sensor. A database of voltage-gated potassium channels was constructed using a representative from each of the Kv subfamilies as the query of a PSI-BLAST search with an E-value of 0.001 until convergence. To create a non-redundant dataset, the Kv channels were clustered at 90% sequence identity [11]. A total of 147 Kv channels were obtained, and their voltage sensors were extracted based on the known motif [12]. Since these were 18 residues in length, an ungapped alignment was obtained (available as a supporting information). The conservation moments for this functional region were calculated at periodicities corresponding to three different secondary structures: the regular *α* helix, the 3_10_ helix and the *β* sheet. Fig. 7 shows the profile of these three conservation moments over the voltage sensor using a block of 10 residues. Two observations emerged from our analysis. First, the conservation moments of the *α* helix and 3_10_ helix rise much above the average conservation (which could assume a value of 1.0 at the maximum) whereas the *β* strand conformation is disfavored. Conservation moments that exceed the maximum possible conservation would reflect a selection for the moment, indicating possible functional significance. Second, the conservation moment of the 3_10_ helix exceeds that of the *α* helix at position 3 which contains the conserved arginine residue of the gating pore. The segment is uncertain in its preference for the *α* helix, especially at the positions containing the conserved positively charged residues. This suggested a dominant preference for the 3_10_ helical conformation over the alpha helix over the length of canonical motif. Surprisingly, crystallographic studies of the voltage-gated potassium channel have determined this region to adopt an unusual transmembrane 3_10_ helix, stretching out inside an *α* helical conformation at the ends of the voltage sensor [13]. This local 3_10_ helical conformation accounted for the energetics of the voltage sensor movement in a hydrophobic lipid membrane environment. The S4 helix (i.e, the voltage sensor) maintained an entire face of spatially oriented positively charged residues, which could interact with conserved acidic residues from other TM helices, forming stabilizing ion pairs. The opposite face of the S4 helix was variable, maintaining a hydrophobic character that would have preferred the *α* helical conformation, if it were not for the voltage sensing motif. Our analysis was able to detect this asymmetry crucial to the gating pore and seemed to provide support for mechanisms of gating that involve the formation of ion pairs with 3_10_ helices in the S4 voltage sensor. It is clear from this example that the conservation moment captured a feature of evolution that would not have been apparent from an examination of residue-by-residue conservation.

## 4 Conclusion

The proposed conservation moment demonstrated its effectiveness in the analysis of the pore helix and the voltage sensor of potassium channels. It was observed that oscillations in conservation moments matched the period of the *α*-helix enabling differential conservation of packing interfaces of the pore helix. In the case of the voltage sensor, the method of conservation moments detected the preference for the rare 3_10_-helix over the *α*-helix. Two conclusions could be made from the above. Differential moments for the periodicities corresponding to different secondary structures would be predictive of the ‘momentous' secondary structure. Second, facially differential conservation within secondary structures (i.e, the existence of a significant conservation moment in the secondary structure) would be diagnostic of regions of functional activity. The profile of conservation moments of a protein sequence calculated using an appropriate profile would be useful in detecting both the spatially asymmetric conservation and the secondary structure preference. It would be a valuable tool in interrogating structure–function relationships in proteins and its potential for the automated detection of functionally important regions in proteins could be explored in the future. The conservation moment embodies an enrichment of the information contained in residue conservation. The implemented algorithm could be applied with little modification to calculate the strength of Fourier components and detect periodicity in the one-dimensional function of any residue property including hydrophobicity and packing. By combining the information and moments of both physical and evolutionary properties, higher-order trends could be found.

## 5 Supporting Information

The software for calculating *C*_1_(*θ*) and supplementary data are available in the following repository: https://github.com/apalania/consMoment.
